# CRO-INSIGHT: Utilization of Implantable Cardioverter Defibrillators in Non-ischemic and Ischemic Cardiomyopathy in a Single Croatian Tertiary Hospital Centre

**DOI:** 10.31083/RCM26349

**Published:** 2025-04-16

**Authors:** Mislav Puljevic, Eugen Ciglenecki, Vedran Pasara, Ivan Prepolec, Mia Dubravcic Dosen, Pero Hrabac, Ana-Marija Brekalo, Martina Lovric Bencic, Miroslav Krpan, Richard Matasic, Borka Pezo-Nikolic, Davor Puljevic, Davor Milicic, Vedran Velagic

**Affiliations:** ^1^Department of Cardiovascular Diseases, University Hospital Centre Zagreb, 10000 Zagreb, Croatia; ^2^Department of Cardiovascular Diseases, University of Zagreb School of Medicine, 10000 Zagreb, Croatia

**Keywords:** implantable cardioverter defibrillator, cardiac resynchronization therapy defibrillator, non ischemic heart failure, survival outcomes, device activation

## Abstract

**Background:**

Implantable cardioverter defibrillators (ICDs) have significantly reduced the incidence of sudden cardiac death in patients with heart failure, particularly those with ischemic heart disease. However, the impact on overall mortality remains controversial, especially in non-ischemic heart failure patients. The Danish Study to Assess the Efficacy of ICDs in Patients with Non-Ischemic Systolic Heart Failure (DANISH) trial and subsequent studies have questioned the efficacy of ICDs in this population, particularly among older patients. The present study aimed to evaluate survival outcomes and predictors in a Croatian cohort of patients with an ICD or cardiac resynchronization therapy defibrillator (CRT-D) device.

**Methods:**

This retrospective cohort study analyzed data from 614 patients who received an ICD or CRT-D device at KBC Zagreb between 2009 and 2018. Patient data, including demographic information, device indication, and clinical parameters, were collected at the time of implantation. Follow-up data were systematically recorded to assess device activation and survival outcomes. Statistical analyses included a detailed descriptive analysis, Kaplan–Meier survival estimates, and Cox regression models.

**Results:**

The cohort consisted predominantly of males (83.4%), with a mean age of 58.7 years. Most had reduced left ventricular ejection fraction (mean 31.4%) and were classified as New York Heart Association (NYHA) class II or III. Over a median follow-up of 48.4 months, 36.6% of patients died. Device activation occurred in 30.3% of patients, with appropriate activation observed in 88.2% of these cases. Cox regression identified age, non-sustained ventricular tachycardia (NSVT), and decompensation history as significant survival predictors.

**Conclusions:**

This study confirmed that appropriate device activation improved survival in patients with an ICD/CRT-D. Age, NSVT, and history of decompensation were key predictors of device activation and survival outcomes. These findings underscore the need for individualized patient assessment when considering inserting ICDs, particularly in non-ischemic heart failure patients. Further research is needed to refine clinical guidelines and optimize patient selection for ICD therapy.

## 1. Introduction 

Over the past two decades, the implantation of implantable cardioverter-defibrillators (ICDs) has been associated with a significant reduction in the incidence of sudden cardiac death and overall mortality among patients with ischemic heart disease. In contrast, no individual study, nor subgroup analysis within studies examining all cardiomyopathy patients, has demonstrated a statistically significant difference between ICD therapy and medical therapy in non-ischemic cardiomyopathy, although there has been a clear trend favoring ICD therapy [1]. It was through a meta-analysis of all studies in 2017 that a statistically significant benefit of ICDs in reducing overall mortality for non-ischemic cardiomyopathy was established [2].

The aforementioned studies did not employ the same concomitant therapies. The only study that achieved statistical significance was the COMPANION trial (Comparison of Medical Therapy, Pacing, and Defibrillation in Heart Failure), where ICDs were combined with cardiac resynchronization therapy (CRT), which most likely significantly contributed to the positive outcome. The study also found no difference between patients with cardiac resynchronization therapy defibrillator (CRT-D) devices and those with cardiac resynchronization therapy pacemaker (CRT-P) devices [3]. Similarly, the Sudden Cardiac Death in Heart Failure Trial (SCD-HeFT) explicitly noted that all patients were on optimal medical therapy at the time, which included beta-blockers, angiotensin-converting enzyme (ACE) inhibitors, and aldosterone antagonists [4]. It is well-known that mortality (both overall and sudden death) has been significantly reduced since the systematic introduction of these medications. Notably, this study demonstrated a significant reduction in mortality in the overall patient group with ICD therapy. However, when observing patients by New York Heart Association (NYHA) class, no difference between ICDs and placebo was found in NYHA class III patients (*p* = 0.3) [4]. The difference was present only in the NYHA class II group. Similarly, when analyzing only the subgroup with non-ischemic cardiomyopathy, although the number of patients was comparable to the ischemic cardiomyopathy group, the benefit of ICDs did not reach statistical significance, despite showing a positive trend [4].

The more recent Danish study (DANISH), with the longest follow-up period (9.5 years) and contemporary optimal medical therapy in the non-ischemic cardiomyopathy group, did not demonstrate a benefit of ICDs in reducing overall mortality compared to medical therapy [1]. However, some benefit was observed in patients under 70 years of age, while no benefit was seen in older patients [5, 6]. In this study, 58% of patients also received resynchronization therapy. The group older than 70 years had a slightly higher rate of CRT therapy (68%), which may have contributed to the reduced benefit of ICDs [7]. On the other hand, the ratio of sudden to non-sudden cardiac death (non-SCD) was lower in younger patients compared to older ones, which may also influence the results of ICD therapy. In the younger population (<70 years), the incidence of sudden cardiac death (SCD) is 1.8, while the incidence of non-SCD is 2.7 events per 100 patient-years. In the population older than 70 years, the incidence of SCD is 1.5, whereas the incidence of non-SCD is significantly higher at 5.4 events per 100 patient-years [7].

Cardiomyopathy is not a homogeneous condition, and risk assessment depends on the type of cardiomyopathy (ischemic/non-ischemic). Even non-ischemic cardiomyopathy is not a uniform group, encompassing dilated, myocarditis, arrhythmogenic dysplasia, restrictive, hypertrophic, and other forms. Increasing evidence suggests that a standardized approach to all patients is inadequate, as reflected in the latest ESC guidelines for sudden death prevention, which have downgraded the indication for primary prophylaxis in non-ischemic cardiomyopathy to a IIa recommendation [8]. This has prompted calls for revisiting guidelines for ICD use in primary prevention in non-ischemic heart failure, since newer treatment of heart failure have been developed since the initial recommendations were established [4, 9].

There is growing evidence that reduced systolic function alone is not a precise predictor of whether ICD therapy will significantly impact mortality across all patients. In addition to age, the presence of myocardial fibrosis on cardiac magnetic resonance (CMR) imaging is now increasingly recognized as critical for assessing the risk of sudden death and the potential benefit of ICD therapy [10, 11, 12]. Most patients with ischemic cardiomyopathy have myocardial fibrosis, whereas this is not the case for non-ischemic cardiomyopathy.

In recent years, new medications, such as sacubitril/valsartan and sodium-glucose co-transporter-2 inhibitors, have been introduced and have resulted in a significant reduction of mortality beyond the benefits of standard medical therapy [13, 14].

In view of the conflicting results of existing studies, particularly in the non-ischemic cardiomyopathy group, the need for better stratification and even individualized therapy has been highlighted.

The aim of this study was to identify the characteristics of patients for whom ICD activation was justified and to determine whether certain clinical or laboratory parameters could better stratify patients who would derive greater benefit from ICD implantation.

## 2. Subjects and Methods

This study was conducted on patients from the University Hospital Centre (UHC) Zagreb (the largest hospital in Croatia) who had an ICD or CRT-D implanted between 2009 and 2018. This period was chosen because, starting in 2009, the number of primary prevention implantations in our institution significantly increased, making it economically feasible to implement adequate primary prevention only from that point onward. It is important to note that UHC Zagreb, as the only national hospital, serves patients from across the Republic of Croatia. Most primary and secondary prevention procedures in Croatia are performed at this centre. This study was retrospective in nature.

Indications for ICD and CRT-D implantation were established according to the ESC guidelines at the time [15]. In almost the entire group, indications were limited to Class I recommendations of the guidelines. We randomly used Medtronic (Medtronic Parkway, Minneapolis, MN, USA), St. Jude (Danny Thomas Place, Memphis, TN, USA), and Biotronik (Biotronik Se & Co., Berlin, Germany) devices. Systolic function was expressed as the ejection fraction determined via echocardiography by a licensed specialist, while the type of cardiomyopathy (ischemic/non-ischemic) was determined through medical history, echocardiography, and coronary angiography (without significant coronary stenoses that would explain poor systolic function).

Each device was implanted by an experienced cardiologist under sterile conditions following professional standards. During the procedure, electrode parameters and final device programming were carried out in collaboration with authorized personnel from the device manufacturer. Follow-up was systematically conducted by the same team, recording all device parameters. In cases where therapy was activated, the reason for activation, the type, and the success of the therapy were documented. All data were entered into an Excel spreadsheet.

This was a retrospective study. Data was collected from archives pertaining to the day of implantation. Device check-ups and registration of changes in clinical parameters or adverse events (device activation, hospitalization, therapy changes) were performed annually or, towards the end of the device’s lifespan, every 3–6 months until device replacement. Follow-ups were conducted in outpatient clinics with the patient present. If the patient did not attend a scheduled follow-up, their status was checked with family members or the relevant state institution of their residence.

Statistical analysis was conducted using two software packages: SPSS v25 (IBM Corp. Released 2017. IBM SPSS Statistics for Windows, Version 25.0. Armonk, NY, USA), licensed to the Faculty of Medicine at the University of Zagreb, and Jamovi v2.5 (Jamovi Project (2024), Jamovi (Version 2.5) [Computer Software]. This software was retrieved from https://www.jamovi.org), which is open-source software.

The threshold for statistical significance (alpha, Type I error) was set at 0.05, and no correction for multiplicity was applied as the number of analyses did not require such an approach. Descriptive statistics for individual variables are presented as mean values with standard deviations (for ratio-scale variables) or as frequencies and counts (for nominal or ordinal-scale variables). Differences between observed groups were analysed using appropriate parametric or non-parametric tests, depending on the type and normality of the distribution of individual variables. Normality of distribution was tested using the Shapiro-Wilk test.

Survival analysis was performed in three ways: (1) a simple analysis of differences between two groups—deceased and surviving patients; (2) for variables that showed statistically significant differences between deceased and surviving patients, a Kaplan-Meier survival analysis was conducted; (3) finally, all variables were included as predictors in a Cox regression survival analysis.

## 3. Results

A total of 786 individuals were included in this study. However, upon reviewer’s insisting on listwise missing cases deletion, the final sample size was 614 subjects. They were predominantly males (N = 512; 83.4%), with less of one-fifth of the participants being female (N = 102; 16.6%). The average age of participants at the time of implantation or inclusion in the study was 58.7 ± 13.3 years, with a median age of 60.0 years and an interquartile range (IQR) of 53.0 to 68.0 years. More than two-fifths of the participants (N = 252; 41.0%) were smokers. The mean left ventricular ejection fraction at the time of device implantation was 31.4 ± 11.6%, with a median and IQR of 30.0 and 25.0 to 35.0%, respectively. According to the NYHA classification, participants mainly belonged to class III (N = 174; 28.3%) or class II (N = 301; 49.0%), with a smaller number of participants in class I (N = 125; 20.4%) or class IV (N = 14; 2.3%) (Table 1).

**Table 1.** **Baseline characteristics of subjects**.

Parameter		
Age [years; M ± SD]		58.7 ± 13.3
Gender [N; male (%)]		512 (83.4%)
Smoker [N; yes (%)]		252 (41.0%)
Ejection fraction [%; M ± SD]		31.4 ± 11.6
NYHA class [N; (%)]		
	I	125 (20.4%)
	II	301 (49.0%)
	III	174 (28.3%)
	IV	14 (2.3%)
Indication [N; (%)]		
	Cardiac arrest	101 (16.4%)
	Low EF	330 (53.7%)
	VT	65 (10.6%)
	NSVT	118 (19.2%)
Concomitant medication [N; (%)]		
	Beta-blocker	565 (92.0%)
	ACEi/ARB	475 (77.4%)
	Amiodaron	227 (37.0%)

SD, standard deviation; NYHA, New York Heart Association; EF, ejection fraction; VT, ventricular tachycardia; NSVT, non-sustained ventricular tachycardia; ACEi, angiotensin-converting enzyme inhibitor; ARB, angiotensin receptor blocker.

All participants had a device implanted between January 2009 and February 2018. The end of the follow-up period was determined to be December 31, 2019. The mean follow-up duration was 52.9 ± 21.5 months, with a median follow-up time of 48.4 months and an IQR of 36.6 to 60.6 months. During the study period, 225 participants (36.6%) expired.

The majority of participants (N = 565; 92.0%) were taking beta-blockers, followed by angiotensin-converting enzyme inhibitors/angiotensin receptor blockers (N = 475; 77.4%), amiodarone (N = 227; 37.0%) (Table 1).

Depending on the indication, either an ICD (N = 478; 77.9%) or a CRT-D (N = 136; 22.1%) device was implanted. ICD devices were mostly (N = 450; 94.1%) single-chamber type devices, while CRT-D devices were mostly (N = 111; 81.6%) dual-chamber type devices.

The indication for device implantation was a previous cardiac arrest (N = 101; 16.4%), reduced ejection fraction (N = 330; 53.7%), ventricular tachycardia (N = 65; 10.6%), or non-sustained ventricular tachycardia (NSVT) (N = 118; 19.2%). All participants had some form of cardiomyopathy, 286 participants (46.6%) had ischemic and 328 participants (53.4%) had non-ischemic cardiomyopathy.

Other pre-implantation parameters, including electrocardiogram (ECG) parameters and comorbidities, are shown in Table 2. The majority of participants had a history of hypertension (65.3%), decompensation (59.4%), and myocardial infarction (39.4%), while diabetes (27.2%) and thyroid diseases (17.8%) were less common. Electrocardiogram parameters included ventricular tachycardia (60.9%), left bundle branch block (34.5%), atrial fibrillation/flutter (30.9%), and ventricular fibrillation (13.5%). Table 3 presents the distribution of patients based on the types of cardiomyopathy observed in the study cohort. The majority of cases are categorized as ischemic or non-ischemic cardiomyopathy, with non-ischemic cardiomyopathy further subdivided into specific types, including dilated cardiomyopathy, arrhythmogenic right ventricular dysplasia, and other less common forms.

**Table 2.** **Preimplantation ECG parameters and comorbidities**.

Parameter [N; yes (%)]	
ECG – Ventricular tachycardia	374 (60.9%)
ECG – Ventricular fibrillation	83 (13.5%)
ECG – Atrial fibrillation/undulation	190 (30.9%)
ECG – Left bundle branch block	212 (34.5%)
History of myocardial infarction	242 (39.4%)
History of decompensation	365 (59.4%)
Hypertension	401 (65.3%)
Diabetes	167 (27.2%)
Thyroid condition	109 (17.8%)

ECG, electrocardiogram.

**Table 3.** **Types of cardiomyopathy**.

Type of CMP	Counts	% of Total
Thyrotoxic	1	0.2%
Toxic	4	0.7%
ARVD	5	0.8%
Non-compaction	9	1.5%
General rhythm	15	2.4%
Dilatative	97	15.8%
Non-ischemic	197	32.1%
Ischemic	286	46.6%

CMP, cardiomyopathy; ARVD, arrhythmogenic right ventricular dysplasia.

**Fig. 1.**
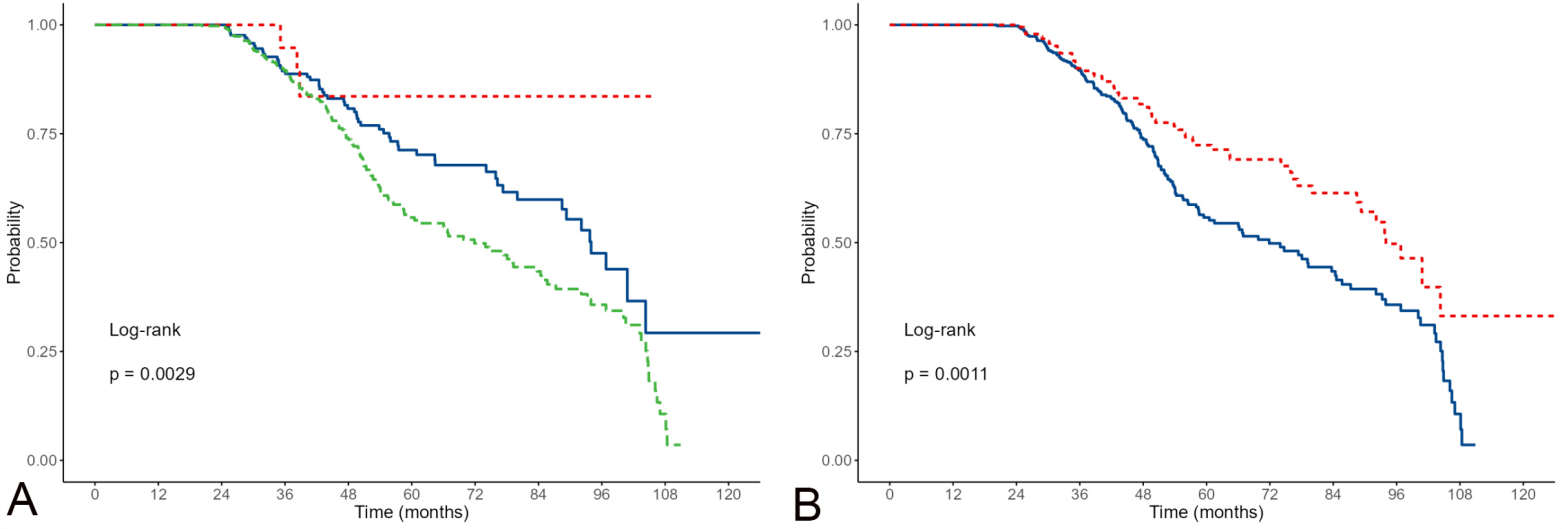
**Kaplan-Meier estimates of survival for appropriate/non-appropriate activations including non-activations**. (A, appropriate are red; inappropriate green; non-activations blue); and appropriate/non-appropriate activations excluding non-activations (B, appropriate activations are red; non-activations blue).

One of the study’s objectives was to thoroughly investigate the appropriateness of device activation in relation to other available parameters in the study. In the majority of participants (N = 428; 69.7%), the device did not activate. Of the remaining 186 participants (30.3%) whose device activated, the activation was appropriate in the vast majority of cases (N = 164; 88.2%), and inappropriate in only 11.8% (N = 22) of participants. Fig. 1 shows the survival results concerning different device activation parameters. There was a statistically significant (*p* < 0.001) difference in survival between participants whose device activated (red on both charts) compared to those whose device did not activate (blue on the charts). The number of participants with inappropriate device activation is shown in the first figure, but these results should be interpreted with caution due to the small number of observations.

In addition to the descriptive data presented in the previous tables, the study was designed to answer several additional questions regarding device activation, the results of which will be described in this section.

When device activation is considered as a binary variable, disregarding appropriateness and looking solely at the presence (N = 186; 30.3%) or absence (N = 428; 69.7%) of activation, outcomes for several key variables were observed. There were no statistically significant differences (*p* = 0.642; chi-square test) in the type of cardiomyopathy (ischemic vs. non-ischemic). This difference became marginally statistically significant (*p* = 0.049) when considering the appropriateness of activation, with a significantly higher percentage of inappropriate activations in non-ischemic cardiomyopathies (5.5%) compared to ischemic ones (1.7%), while for appropriate activations, the situation is reversed with 28.7% appropriate activations in ischemic patients and 26.5% in non-ischemic ones. The proportion of participants without activation is similar between the 2 groups (69.6% in ischemic vs. 68.0% non-ischemic cases). A statistically significant differences were also present for type of prevention (primary vs. secondary cases). When considering only the presence of activation, the proportion of activation was higher in individuals with secondary prevention (39.7% vs. 27.2%; *p* = 0.004; chi-square test). When considering the appropriateness of activation, inappropriate activations were significantly higher (5.0%) in primary prevention compared to secondary prevention participants (0.0%). For appropriate activations, the situation was reversed with 39.7% appropriate activations in secondary and 23.5% in primary prevention participants.

When only the appropriateness of activation is considered as an outcome, either appropriate (N = 169; 88.0%) or inappropriate (N = 23; 12.0%), the profiles of both groups of participants are shown in the Table 4. The statistical significance of the differences is shown in the “*p*” column. Appropriate activation was found to be statistically significant.

**Table 4.** **Characteristics of subjects according to appropriateness of device activation**.

Parameter		Appropriate (N = 169)	Inappropriate (N = 23)	*p*
Age [years; M ± SD]		57.6 ± 15.0	50.3 ± 12.9	<0.027
Gender [N; male (%)]		143 (84.6%)	21 (91.3%)	0.591
Smoker [N; yes (%)]		67 (39.6%)	9 (39.1%)	0.962
Ejection fraction [%; M ± SD]		33.0 ± 12.6	31.5 ± 8.9	0.590
NYHA class [N; (%)]				
	I	43 (25.4%)	4 (17.4%)	0.138
	II	83 (49.1%)	15 (65.3%)	
	III	42 (24.9%)	3 (13.0%)	
	IV	1 (0.6%)	1 (4.3%)	
Indication [N; (%)]				
	Cardiac arrest	37 (21.9%)	0 (0.0%)	0.027
	Low EF	62 (36.7%)	17 (73.9%)	<0.001
	VT	32 (18.9%)	2 (8.7%)	0.227
	NSVT	39 (23.1%)	4 (17.4%)	0.539
Concomitant medication [N; (%)]				
	Beta-blocker	155 (91.7%)	23 (100.0%)	0.314
	ACEi/ARB	126 (74.6%)	19 (82.6%)	0.559
	Amiodaron	70 (41.4%)	5 (21.7%)	0.070
ECG – Ventricular tachycardia		137 (81.1%)	11 (47.8%)	<0.001
ECG – Ventricular fibrillation		30 (17.8%)	0 (0.0%)	0.058
ECG – Atrial Fibrillation/undulation		45 (26.6%)	13 (56.5%)	0.003
ECG – Left bundle branch block		53 (31.4%)	3 (13.0%)	0.117
History of myocardial infarction		67 (39.6%)	5 (21.7%)	0.096
History of decompensation		96 (56.8%)	12 (52.2%)	0.674
Hypertension		99 (58.6%)	14 (60.9%)	0.834
Diabetes		39 (23.1%)	8 (34.8%)	0.221
Thyroid condition		36 (21.3%)	3 (13.0%)	0.517
Cardiomyopathy [N; ischemic (%)]		82 (48.5%)	5 (21.7%)	0.015
Prevention [N; (%)]				
	Primary	109 (64.5%)	23 (100.0%)	<0.001
	Secondary	60 (35.5%)	0 (0.0%)	

The statistical significance of the differences, as indicated in the “*p*” column, reveals that appropriate activation was statistically significant in subjects who were older, had a history of cardiac arrest, lower ejection fraction (EF), took amiodarone, had ventricular tachycardia or ventricular fibrillation on ECG, a history of myocardial infarction, or ischemic cardiomyopathy.

To additionally analyse the impact of various parameters on the appropriateness and/or presence of activation, binary and multinomial regression analyses were conducted.

In the multinomial regression analysis model using the forward stepwise method, i.e., when activation was considered by the model as non-activated/appropriate/inappropriate, none of the models reached an R^2^ value above 0.05, so these models will not be further considered. However, in the binary logistic regression model where the outcome was the appropriateness of activation (appropriate/inappropriate; N = 192), the forward stepwise method found models with the following number of predictors:

(1) Ventricular tachycardia in ECG (R^2^ = 0.106),

(2) Ventricular tachycardia in ECG, prevention (R^2^ = 0.244),

(3) Ventricular tachycardia in ECG, prevention, atrial fibrillation/undulation in ECG (R^2^ = 0.310).

Ventricular tachycardia in ECG, prevention, atrial fibrillation/undulation in ECG, Left bundle branch block in ECG (R^2^ = 0.371). We began the survival analysis with the most straight-forward method, examining the differences between surviving and deceased patients across all available parameters in the study. The results are presented in Table 5.

**Table 5.** **Characteristics of deceased vs. alive subjects at the end of the follow-up period**.

Parameter		Alive (N = 389)	Deceased (N = 225)	*p*
Age [years; M ± SD]		56.1 ± 13.1	63.2 ± 12.3	<0.001
Gender [N; male (%)]		321 (82.5%)	191 (84.9%)	0.447
Smoker [N; yes (%)]		167 (42.9%)	85 (37.8%)	0.211
Ejection fraction [%; M ± SD]		32.6 ± 12.0	29.3 ± 10.6	<0.001
NYHA class [N; (%)]				
	I	95 (24.4%)	30 (13.3%)	<0.001
	II	200 (51.4%)	101 (44.9%)	
	III	87 (22.4%)	87 (38.7%)	
	IV	7 (1.8%)	7 (3.1%)	
Indication [N; (%)]				
	Cardiac arrest	62 (15.9%)	39 (17.3%)	0.653
	Low EF (<35%)	199 (51.2%)	131 (58.2%)	0.091
	VT	46 (11.8%)	19 (8.4%)	0.190
	NSVT	82 (21.1%)	36 (16.0%)	0.124
Concomitant medication [N; (%)]				
	Beta-blocker	356 (91.5%)	209 (92.9%)	0.545
	ACEi/ARB	295 (75.8%)	180 (80.0%)	0.235
	Amiodaron	130 (33.4%)	97 (43.1%)	0.017
ECG – Ventricular tachycardia		244 (62.7%)	130 (57.8%)	0.226
ECG – Ventricular fibrillation		53 (13.6%)	30 (13.3%)	0.919
ECG – Atrial fibrillation/undulation		112 (28.8%)	78 (34.7%)	0.129
ECG – Left bundle branch block		128 (32.9%)	84 (37.3%)	0.266
History of myocardial infarction		143 (36.8%)	99 (44.0%)	0.077
History of decompensation		212 (54.5%)	153 (68.0%)	0.001
Hypertension		242 (62.2%)	159 (70.7%)	0.034
Diabetes		90 (23.1%)	77 (34.2%)	0.003
Thyroid condition		67 (17.2%)	42 (18.7%)	0.652
Cardiomyopathy [N; ischemic (%)]		160 (41.1%)	126 (56.0%)	<0.001

**Fig. 2.**
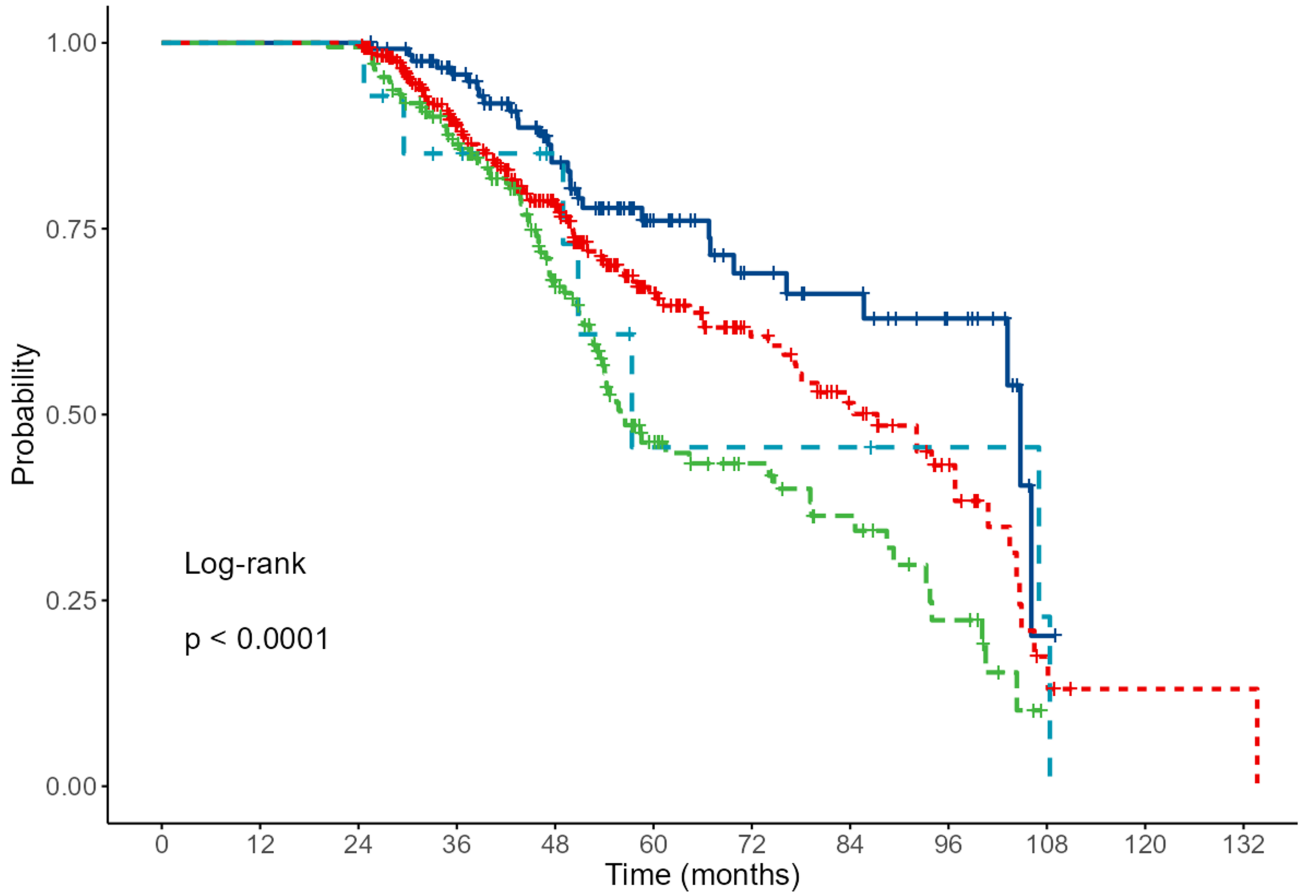
**Results of Kaplan-Meier analysis for NYHA**. Grade 1 (dark blue), 2 (red), 3 (green) and 4 light blue (*p* < 0.001; Log-rank test).

As the next step, we applied the Kaplan-Meier method (Fig. 2) to nominal or ordinal variables that showed statistical significance in the previous table. The results are presented in the graphs below.

Finally, the Cox regression survival analysis resulted in 3 models: the first model included only age as a predictor, the second model included both age and non-sustained ventricular tachycardia, and the third model adds a history of decompensation as an additional predictor (Table 6).

**Table 6.** **Results of the Cox regression analysis**.

Model	Variables	B	SE	Wald	*p*	Exp(B)	95% CI
1	age	0.037	0.006	39.705	0	1.038	1.026–1.050
2	age	0.037	0.006	39.190	0	1.038	1.026–1.050
	ventricular tachycardia in ECG	–0.791	0.141	31.626	0	0.454	0.344–0.597
3	age	0.037	0.006	36.370	0	1.037	1.025–1.050
	ventricular tachycardia in ECG	–0.794	0.140	31.959	0	0.452	0.343–0.595
	history of decompensation	0.464	0.144	10.429	0.001	1.591	1.200–2.109
4	age	0.038	0.006	38.034	0	1.038	1.026–1.051
	indication of VT	0.808	0.205	15.505	0	2.243	1.501–3.354
	ventricular tachycardia in ECG	–0.984	0.153	41.557	0	0.374	0.277–0.504
	history of decompensation	0.574	0.148	15.091	0	1.775	1.329–2.371

## 4. Discussion

The first 5 studies on primary mortality prevention in patients with cardiomyopathy mostly demonstrated a positive impact of ICD on mortality (MADIT I and II, MUSTT). However, the CABG-Patch and CAT studies did not achieve statistical significance, though the CAT study showed a positive trend in the ICD group. It is believed that the potential reason for this is the positive impact of revascularization, while in the CAT study, which investigated non-ischemic cardiomyopathy, mortality was significantly lower than expected (5.6%, expected 30%). Furthermore, the number of patients in the study was relatively small. To increase the study’s power by increasing the number of patients, a meta-analysis was conducted. This meta-analysis showed a statistically significant 34% reduction in mortality in the ICD group. However, when the non-randomized MUSTT study was excluded, there was no statistical significance in overall mortality between groups (*p* = 0.124), though a difference was observed when considering arrhythmic death alone (*p* < 0.01).

Subsequently, 5 newer studies were published (AMIOVIRT, DEFINITE, COMPANION, DINAMIT, and SCD-HeFT). The first 2 studies focused solely on non-ischemic cardiomyopathy, while the other 3 examined either ischemic cardiomyopathy or cardiomyopathy in general. In the studies on non-ischemic cardiomyopathy, there was no statistically significant difference in mortality between the group receiving optimal medical therapy and the ICD group, although the DEFINITE study showed a trend toward lower mortality in the ICD group (13.8% vs. 8.1%) with a 41% relative risk (RR) reduction, albeit without statistical significance (*p* = 0.081). It should be noted that follow-up in these 2 studies was up to 3 years.

The COMPANION study was primarily based on the impact of resynchronization therapy on mortality, so all patients had a prolonged QRS complex (>120 ms). After a relatively short follow-up of 1 year, the CRT-D group showed a significant reduction in mortality (*p* = 0.004) with an RR reduction of 36%. The mortality reduction in the CRT-P group was 24% (*p* = 0.063), and there was no significant difference in mortality between the CRT-P and CRT-D groups.

The last and largest study, SCD-HeFT, with a follow-up of 4 years, showed a significant reduction in mortality in the ICD group (*p* = 0.007) with a 24% RR reduction. Mortality in the ICD group was 22%, compared to 28% in the amiodarone group and 29% in the control group. However, a subgroup analysis by NYHA classification showed significant differences only in the NYHA II group (*p* < 0.001), while there were no differences in the NYHA III group (*p* = 0.362, hazard ratio 0.84–1.61). Subgroup analysis based on the type of cardiomyopathy also showed some differences. A significant reduction in mortality was found in ischemic cardiomyopathy (*p* = 0.049), while in the non-ischemic cardiomyopathy group, the difference did not reach significance (*p* = 0.062), although there was a positive trend. It should be emphasized that the number of patients in both cardiomyopathy groups was similar.

In the group of patients with the lowest baseline risk, ICD reduced the risk of sudden death by 88% and total mortality risk by 54%, while in the group with the highest baseline risk, sudden death mortality was reduced by 24%, and total mortality by only 2%. A meta-analysis of all studies showed a 25% reduction in overall mortality RR in the ICD group (*p* = 0.003). Overall mortality in the ICD group was 18.5%, compared to 26.4% in the control group.

The Danish study on the efficacy of ICDs in non-ischemic cardiomyopathy did not show a reduction in overall mortality in the ICD group compared to the group receiving optimal medical therapy. The average age of patients was 63 years (21–84), with an average follow-up period of 5.6 years. Sub-analysis revealed that in the group <70 years of age, ICD use was associated with a mortality reduction, while in the group >70 years, ICD use did not affect mortality. It should be noted that, in addition to optimal medical therapy, 58% of patients in the ICD group also received resynchronization therapy. The percentage of resynchronization therapy was higher in the >70 age group (68%), which might partially explain the reduced efficacy of ICDs in this group.

In the <70 years group, sudden death occurred in 1.8 cases and non-sudden death in 2.7 cases per 100 patient-years, while in the >70 years group, sudden death occurred in 1.6 cases and non-sudden death in 5.4 cases per 100 patient-years. The difference between these 2 groups was significant (*p* = 0.011). Inappropriate shocks were recorded in 5.9% of patients.

Compared to the Danish study, the population in our study was younger (58.7 years) with a significantly smaller proportion of patients older than 70 years and a slightly shorter follow-up period (4.5 years). Most patients were on optimal medical therapy, with a relatively high percentage also receiving amiodarone (37.0%). This may be related to the initially relatively high incidence of atrial fibrillation/flutter and attempts to control ICD activation during a period when atrial fibrillation, ablation and ventricular tachycardia ablation for structural heart disease were not widely available in our country. The proportion of patients in NYHA class III was slightly higher than in other primary prevention studies. ICD activation did not occur in 69.7% of patients, a slightly lower percentage than in other studies, which could be explained by the inclusion of more severe patients, i.e., a higher proportion of those in NYHA class III, and slightly longer follow-up. In the SCD-HeFT study, mortality in the NYHA III group was almost double that in the NYHA II group.

Among patients with device activation in our study, 12.0% of activations were inappropriate (3.7% of the total population with implanted ICDs), which is similar to the Danish study (5.9%). Inappropriate shocks were mostly due to supraventricular rhythm disorders (AF, atrial flutter, SVT, sustained ventricular tachycardia) and, in fewer cases, technical issues, predominantly involving the lead (fracture, electromagnetic interference, inadequate grounding). All causes were successfully resolved (AF, atrial flutter, or SVT ablation, atrioventricular (AV) node ablation, lead replacement) without further adverse events.

Inappropriate ICD activation was significantly more common in non-ischemic cardiomyopathy (5.5% vs. 1.7%), while the opposite was true for appropriate activations (26.5% vs. 28.7%). When examining the profile of patients with inappropriate activations, it was significantly more common among those who initially did not have ventricular rhythm disturbances, with the indication being solely reduced ejection fraction. Furthermore, inappropriate activation was significantly lower among patients taking amiodarone and those with initial ECG findings of ventricular tachycardia (VT)/ventricular fibrillation (VF) or a history of myocardial infarction. These patients were initially prescribed amiodarone, which likely contributed to fewer inappropriate activations (suppression of supraventricular arrhythmias and slowing of SVT/VT below detection thresholds). Conversely, patients with reduced EF alone were not prescribed antiarrhythmic therapy initially and only received it after inappropriate activation due to AF/flutter, supported by the fact that inappropriate activations were more frequent in patients with recorded AF/flutter.

Patients with ischemic cardiomyopathy were more likely to have severe arrhythmias and were thus prescribed antiarrhythmic therapy earlier, which might have reduced the frequency of arrhythmias and ventricular response below detection thresholds.

Mortality in our study was 36.6%. In the ischemic cardiomyopathy group, 46.6% of patients died, while significantly fewer patients died in the non-ischemic cardiomyopathy group (28.5%). Deceased patients were significantly older, had more severe cardiomyopathy (lower EF, higher NYHA class), and ischemic cardiomyopathy. In the Danish study of non-ischemic cardiomyopathy, mortality was only slightly lower (21.6%) compared to our study, which could be explained by the higher proportion of patients receiving concomitant resynchronization therapy (58% compared to 22.1% in our study). Higher mortality among older patients and those with poorer functional status were observed not only in the Danish study but also in meta-analyses of all primary prevention studies.

In line with these findings, our Cox regression analysis demonstrated that combined predictors of mortality despite ICD implantation were age and a history of heart failure. The presence of NSVT, which also contributes to increased mortality, is harder to explain. However, NSVT in this group could indicate more severe structural heart disease or represent a confounding factor not considered in this study (e.g., side effects of certain antiarrhythmic therapies).

It is unquestionable that ICD reduces sudden death, but there is increasing evidence that better patient stratification is needed to maximize the benefits of ICD therapy. Candidates for ICD implantation should be those with a higher risk of sudden death and lower risk of non-arrhythmic mortality. ICD implantation involves both acute and chronic complications in nearly 20% of patients. According to current guidelines, more than 70% of patients recommended for ICD implantation did not require ICD therapy. Therefore, they did not benefit from this therapy but were exposed to its adverse effects. Furthermore, in most studies, mortality with ICD implantation ranged from 15–20%.

In light of new heart failure drug therapies introduced in recent years, which have demonstrated reduced overall mortality on top of standard therapies, better risk stratification would be desirable, particularly for ischemic and especially non-ischemic cardiomyopathy. There is increasing evidence that myocardial fibrosis detected by cardiac magnetic resonance imaging (MRI) indicates high risk status regardless of systolic function. Patients with ischemic cardiomyopathy generally exhibit significant fibrosis, while this is not typically the case in non-ischemic cardiomyopathy. In a meta-analysis of non-ischemic cardiomyopathy patients [16], the annual rate of malignant arrhythmias was 6.9% in patients with myocardial fibrosis, compared to 1.6% in those without fibrosis, regardless of systolic function (EF> or <35%). It was also found that the annual rate of malignant arrhythmias was 7.3% in patients with EF 36–50% if ventricular fibrosis was present on cardiac MRI.

With newer medical and invasive heart failure therapies, an aging population with more comorbidities, and advanced imaging, risk stratification studies based solely on systolic function may no longer be adequate. Until new studies are published (some involving cardiac MRI are ongoing), current guidelines remain in place. However, the indication for ICD therapy should still be individualized, especially for non-ischemic cardiomyopathy. Consideration should be given to whether ICD implantation is justified in older patients (>65–70 years) with lower functional classes (NYHA III or IV) and signs of heart failure. Similarly, it is worth considering whether prophylactic ICD implantation is necessary for patients with an EF <35% but without myocardial fibrosis on cardiac MRI, who are in good functional status without signs of heart failure and without significant ventricular arrhythmias on Holter monitoring.

## 5. Conclusions

Although ICDs and CRT-D devices are effective in sudden cardiac death, their impact on overall mortality depends on multiple factors, including patient age and type of heart disease. This study suggests that younger patients benefit more from the implantation of the device than the older patient population. In elderly patients >70 years of age, device placement should be more carefully considered taking into account the specific clinical circumstances. The long-term effect of these devices needs to be further investigated and optimal therapeutic strategies adapted to different groups of patients need to be developed. These results emphasize the importance of a personalized approach in cardiology patients, and the constant adaptation of clinical guidelines to ensure the best outcome for the patient.

This study had several limitations. The study was retrospective, and thus limited to the predefined available parameters. Additional parameters could not be observed as this would result in incomplete findings. The study did not include a control group, as all patients had an ICD implanted. Furthermore, the cause of death (sudden or non-sudden) was unknown, though this was less relevant since all patients had an ICD implanted. Longer follow-up would provide a better assessment of the adequacy of therapy, as some studies achieved significance only after several years of follow-up. However, we believe that these limitations are not critical and do not significantly impact the results or the conclusions drawn from the findings.

## Availability of Data and Materials

All raw data reported in this paper will also be shared by the lead contact upon request.

## References

[1] Køber L, Thune JJ, Nielsen JC, Haarbo J, Videbæk L, Korup E (2016). Defibrillator Implantation in Patients with Nonischemic Systolic Heart Failure. *The New England Journal of Medicine*.

[2] Akel T, Lafferty J (2017). Implantable cardioverter defibrillators for primary prevention in patients with nonischemic cardiomyopathy: A systematic review and meta-analysis. *Cardiovascular Therapeutics*.

[3] Bristow MR, Saxon LA, Boehmer J, Krueger S, Kass DA, De Marco T (2004). Cardiac-resynchronization therapy with or without an implantable defibrillator in advanced chronic heart failure. *The New England Journal of Medicine*.

[4] Bardy GH, Lee KL, Mark DB, SCD-HeFT Pilot Investigators (2020). Sudden Cardiac Death in Heart Failure Trial (SCD-HeFT). *American College of Cardiology*.

[5] Theuns DA, Verstraelen TE, van der Lingen ACJ, Delnoy PP, Allaart CP, van Erven L (2023). Implantable defibrillator therapy and mortality in patients with non-ischaemic dilated cardiomyopathy: An updated meta-analysis and effect on Dutch clinical practice by the Task Force of the Dutch Society of Cardiology. *Netherlands Heart Journal: Monthly Journal of the Netherlands Society of Cardiology and the Netherlands Heart Foundation*.

[6] Elming MB, Nielsen JC, Haarbo J, Videbæk L, Korup E, Signorovitch J (2017). Age and Outcomes of Primary Prevention Implantable Cardioverter-Defibrillators in Patients With Nonischemic Systolic Heart Failure. *Circulation*.

[7] Al-Khatib SM, Pokorney SD (2017). Primary Prevention Implantable Cardioverter Defibrillators in Patients With Nonischemic Cardiomyopathy: Diminishing Returns With Advancing Age?. *Circulation*.

[8] Zeppenfeld K, Tfelt-Hansen J, de Riva M, Winkel BG, Behr ER, Blom NA (2022). 2022 ESC Guidelines for the management of patients with ventricular arrhythmias and the prevention of sudden cardiac death. *European Heart Journal*.

[9] Yancy CW, Jessup M, Bozkurt B, Butler J, Casey DE, WRITING COMMITTEE MEMBERS (2013). 2013 ACCF/AHA guideline for the management of heart failure: a report of the American College of Cardiology Foundation/American Heart Association Task Force on practice guidelines. *Circulation*.

[10] Osman J, Tan SC, Lee PY, Low TY, Jamal R (2019). Sudden Cardiac Death (SCD) - risk stratification and prediction with molecular biomarkers. *Journal of Biomedical Science*.

[11] Freitas P, Ferreira AM, Arteaga-Fernández E, de Oliveira Antunes M, Mesquita J, Abecasis J (2019). The amount of late gadolinium enhancement outperforms current guideline-recommended criteria in the identification of patients with hypertrophic cardiomyopathy at risk of sudden cardiac death. *Journal of Cardiovascular Magnetic Resonance: Official Journal of the Society for Cardiovascular Magnetic Resonance*.

[12] Brown PF, Miller C, Di Marco A, Schmitt M (2019). Towards cardiac MRI based risk stratification in idiopathic dilated cardiomyopathy. *Heart (British Cardiac Society)*.

[13] Pontremoli R, Borghi C, Perrone Filardi P (2021). Renal protection in chronic heart failure: focus on sacubitril/valsartan. *European Heart Journal. Cardiovascular Pharmacotherapy*.

[14] Chen J, Jiang C, Guo M, Zeng Y, Jiang Z, Zhang D (2024). Effects of SGLT2 inhibitors on cardiac function and health status in chronic heart failure: a systematic review and meta-analysis. *Cardiovascular Diabetology*.

[15] Priori SG, Blomström-Lundqvist C, Mazzanti A, Blom N, Borggrefe M, Camm J (2015). 2015 ESC Guidelines for the management of patients with ventricular arrhythmias and the prevention of sudden cardiac death: The Task Force for the Management of Patients with Ventricular Arrhythmias and the Prevention of Sudden Cardiac Death of the European Society of Cardiology (ESC) Endorsed by: Association for European Paediatric and Congenital Cardiology (AEPC). *European Heart Journal*.

[16] Di Marco A, Anguera I, Schmitt M, Klem I, Neilan TG, White JA (2017). Late Gadolinium Enhancement and the Risk for Ventricular Arrhythmias or Sudden Death in Dilated Cardiomyopathy: Systematic Review and Meta-Analysis. *JACC. Heart Failure*.

